# Supplementary figures and images for: Transcranial Magnetic Stimulation Attenuates Dyskinesias and FosB and c-Fos Expression in a Parkinson’s Disease Model
Source: Brain Sci. 2024 Nov 29;14(12):1214. doi: 10.3390/brainsci14121214 (PMC11674860; doi:10.3390/brainsci14121214)

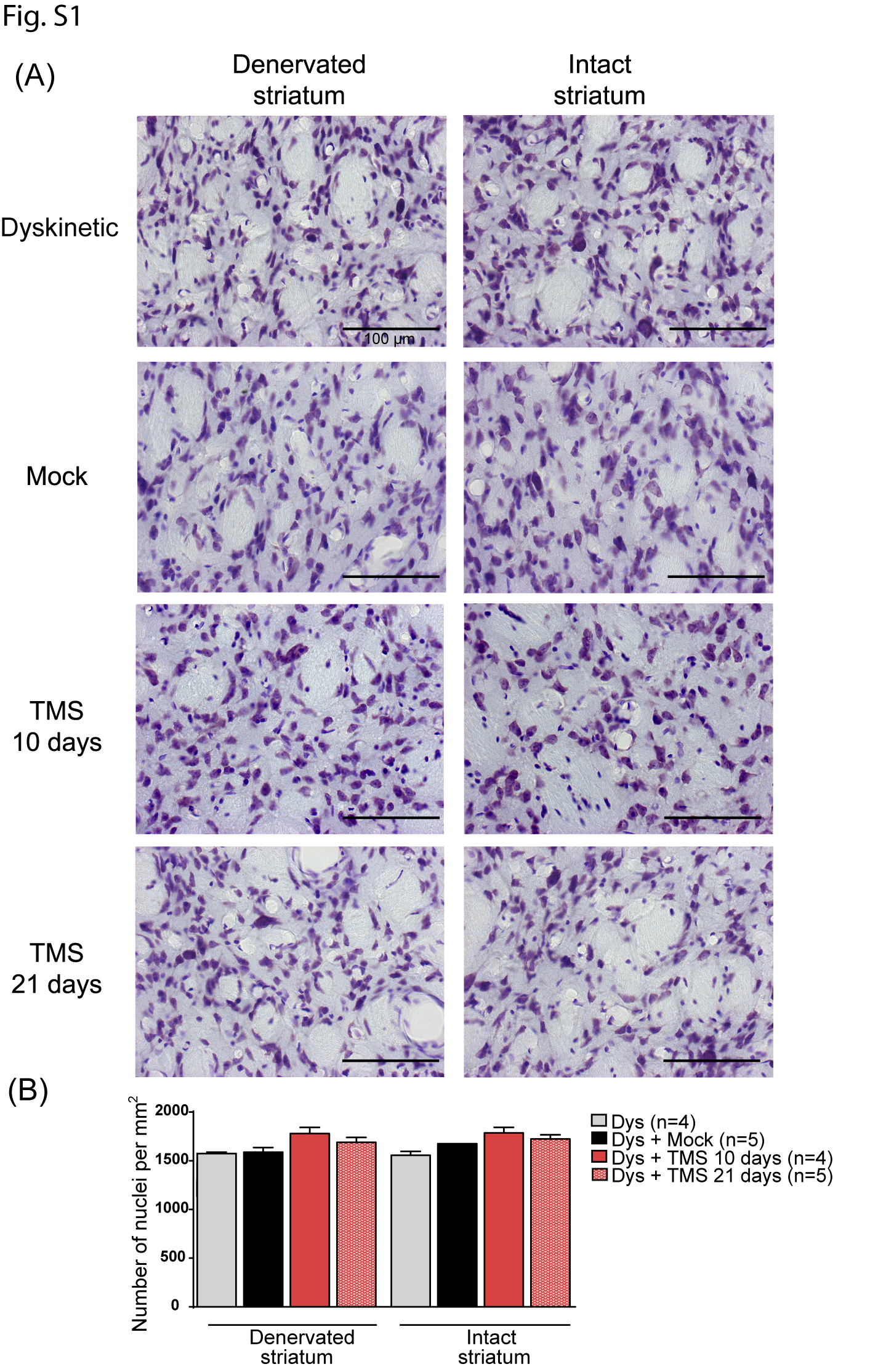

Supplement: Supplementary file 1 [file brainsci-14-01214-s001.zip › brainsci-3314086-supplementary/Fig S1.tif]

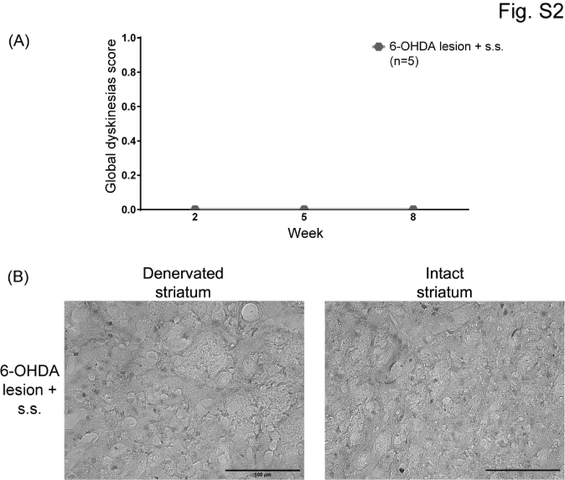

Supplement: Supplementary file 1 [file brainsci-14-01214-s001.zip › brainsci-3314086-supplementary/Fig S2.v2.tiff]

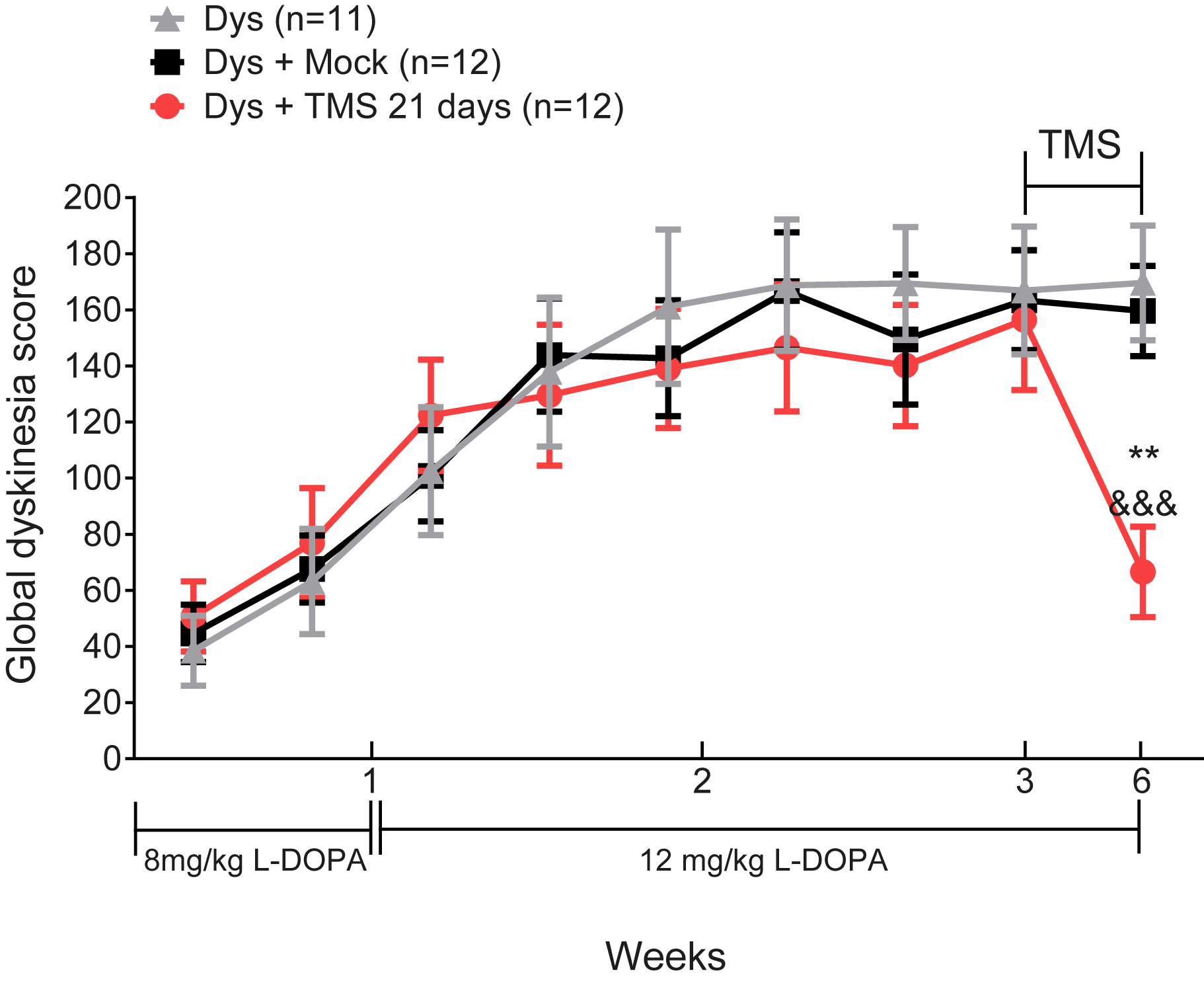

Supplement: Supplementary file 1 [file brainsci-14-01214-s001.zip › brainsci-3314086-supplementary/Fig S3.tif]
